# Unveiling Cerebral Leishmaniasis: parasites and brain inflammation in *Leishmania donovani* infected mice

**DOI:** 10.1038/s41598-017-09085-5

**Published:** 2017-08-16

**Authors:** Guilherme D. Melo, Sophie Goyard, Laurence Fiette, Alexandre Boissonnas, Christophe Combadiere, Gisele F. Machado, Paola Minoprio, Thierry Lang

**Affiliations:** 1 Institut Pasteur, Laboratoire des Processus Infectieux à Trypanosomatidés, Département Infection et Epidémiologie, 25-28 rue du Dr Roux, 75724 Cedex 15, Paris France; 2 UNESP – Univ Estadual Paulista, Faculdade de Medicina Veterinária, Laboratório de Patologia Aplicada (LApap), Rua Clóvis Pestana 793, 16050-680 Araçatuba, São Paulo Brazil; 3 Institut Pasteur, Unité d’Histopathologie Humaine et Modèles Animaux, Département Infection et Epidémiologie, 25-28 rue du Dr Roux, 75724 CEDEX 15, Paris France; 4 Sorbonne Universités, UPMC Univ Paris 06, Inserm, UMR 1135, CNRS, ERL 8255, Centre d’Immunologie et des Maladies Infectieuses (CIMI-Paris), 91 Boulevard de l’Hôpital, 75013 Paris, France; 5 Present Address: Institut Pasteur, Centre d’Innovation et Recherche Technologique, Paris, France

**Keywords:** Inflammation, Parasite host response, Neuroimmunology

## Abstract

Visceral leishmaniasis (VL) is a systemic disease with multifaceted clinical manifestations, including neurological signs, however, the involvement of the nervous system during VL is underestimated. Accordingly, we investigated both brain infection and inflammation in a mouse model of VL. Using bioluminescent *Leishmania donovani* and real-time 2D-3D imaging tools, we strikingly detected live parasites in the brain, where we observed a compartmentalized dual-phased inflammation pattern: an early phase during the first two weeks post-infection, with the prompt arrival of neutrophils and Ly6C^high^ macrophages in an environment presenting a variety of pro-inflammatory mediators (IFN-γ, IL-1β, CXCL-10/CXCR-3, CCL-7/CCR-2), but with an intense anti-inflammatory response, led by IL-10; and a re-inflammation phase three months later, extremely pro-inflammatory, with novel upregulation of mediators, including IL-1β, TNF-α and MMP-9. These new data give support and corroborate previous studies connecting human and canine VL with neuroinflammation and blood-brain barrier disruption, and conclusively place the brain among the organs affected by this parasite. Altogether, our results provide convincing evidences that *Leishmania donovani* indeed infects and inflames the brain.

## Introduction

The brain has been classically considered an immunologically privileged organ, due to the blood-brain barrier (BBB), to the absence of lymphatic vessels and to the reduced expression of MHC-II^1^. Nevertheless, the idea of a completely isolated brain is changing^2^ and the involvement of this organ during inflammation and infectious diseases has been the focus of numerous studies^3, 4^.

Brain inflammation can occur aseptically, such as in stroke^5^ or after peripheral administration of lipopolysaccharide (LPS)^3^. However, several pathogens are able to cross the cerebral barriers and invade the brain, such as rabies virus, *Listeria monocytogenes, Neisseria meningitidis, Cryptococcus neoformans* and *Trypanosoma brucei* ^4, 6^. Convergent case reports suggest that intracellular parasites, which includes *Leishmania* sp^7–9^, might also be able to penetrate the brain, but the mechanisms underlying its entrance into the nervous environment and the ensuing effects are still poorly understood.

Leishmaniasis is a neglected disease caused by different protozoan species of the *Leishmania* genus (Kinetoplastida, Trypanosomatidae) with 0.2–0.4 million new cases/year^10^. Visceral leishmaniasis (VL) is an anthropozoonosis caused by parasites from the *Leishmania donovani* complex: *L. infantum* (syn. *chagasi*), zoonotic, localized mainly in the Americas and in the Mediterranean basin, with dogs representing the main urban reservoir of the disease; and *L. donovani*, anthroponotic, present in Asia and Africa^11, 12^.

VL mostly affects the organs with cells of the mononuclear phagocyte system, such as the liver, spleen, bone marrow and lymph nodes^10^. Although the existence of several reports concerning systemic clinical signs of VL, such as fever, anemia, weight loss, skin lesions, renal disease, ocular alterations^12^, certain studies have related the occurrence of a cerebral form of VL, when neurological injuries and brain inflammation were described in both humans and dogs. Nevertheless, the involvement of the nervous system during VL has certainly been underestimated.

Indeed, peripheral neuropathy and cranial nerves dysfunction have been the most common neurological clinical signs observed in patients with VL in Sudan^13, 14^. Other reports include also neurological tremors and meningitis^15–17^, and in most cases, treatment with anti-leishmanial drugs improve the symptoms^14, 16^. Strikingly, association of VL and Guillain-Barré syndrome is also a possibility^18, 19^, and factors such as malnutrition, immunosuppression, co-infections, may increase the risks of nervous system implication^20–22^. With regards of dogs, neurological manifestations of VL are even more variable, which may include seizures, paresis, cranial nerves dysfunction, meningoencephalitis and hemorrhagic stroke^8, 23–27^.

In view of the paucity of evidences about the presence of the parasite within the central nervous system (CNS), and the actual involvement of the brain during VL, we propose an animal model to analyze the infection dynamics in the brain and to quantify the parasite load in living animals. The proposed model is pioneer to study the brain effects during VL since it allows the evaluation of both infection and inflammatory response in the same animals, widening the possibilities of understanding the pathogenesis of VL.

Following the establishment of brain infection, our objectives were to study the inflammatory process in the brain, to identify if the infected brain is a pro-inflammatory and a chemoattractive environment, which could favor the recruitment of leukocytes, and to characterize the main cell populations involved, including blood-derived macrophages and microglia. Consequently, using virulent bioluminescent *L. donovani* parasites and real time *in vivo* imaging combined with RT-qPCR, we evaluated the presence and the kinetics of parasite migration into the brain, comparing with the peripheral targets of the parasite (liver, spleen, bone marrow). Additionally, we monitored the inflammatory response in these organs, evaluating the populations of inflammatory cells, the gene expression of selected chemokines, chemokines receptors, cytokines, and enzymes of the matrix metalloproteinases family in this mouse model of VL. Our results revealed that *L. donovani* is indeed able to attain and to persist in the brain, triggering a dual-phased inflammatory reaction which is correlated to the parasite load in the periphery as well as to the pattern of the observed immune responses. The implications of those findings are discussed.

## Results

### Leishmania donovani is able to infect and persist in the brain

**Figure 1:**
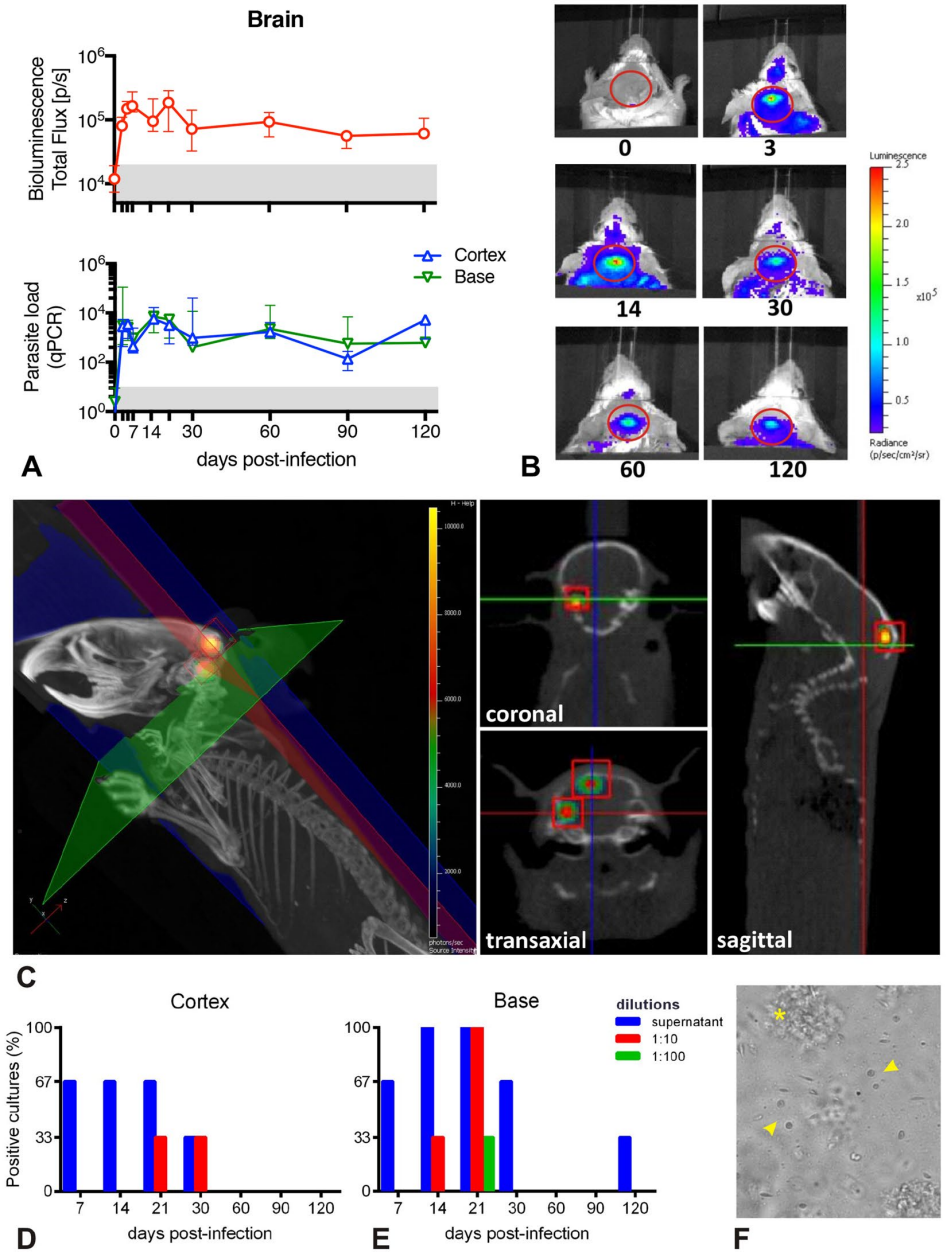
Parasite detection in the brain of BALB/c mice infected with *Leishmania donovani*. (**A**) Kinetics of the parasite load in the brain detected by bioluminescence (upper panel) and in the cortex and base detected by RT-qPCR (bottom panel). The grey area corresponds to background signals. Data are represented as median and the interquartile range. (**B**) Representative images illustrating the 2D-bioluminescence analysis of the parasite detection in the brain at specific time points post infection. The regions of interest (ROIs) corresponding to the brain are represented as red circles. (**C**) *In vivo* tridimensional micro-computed tomography to localize the bioluminescence signals within the brain. Representative tridimensional reconstruction of an infected mouse skeleton at 120 days post infection exhibiting the x-axis (blue), y-axis (green) and z-axis (red), and two bioluminescent foci (red cubes). The cross-sectional images exhibit the coronal (z-axis), transaxial (y-axis) and sagittal (x-axis) views and allow the localization of both bioluminescent foci within the cranial cavity (red squares). See also Movie S1. (**D**,**E**) Parasite isolation from brain tissue. Percentage of positive parasite cultures from the cortex (**D**) and from the base (**E**) of infected BALB/c mice brains, according to different dilutions and time points (n = 3/time point). (**F**) Representative image of a parasite culture from a cortex at day 7 post infection, where it is possible to observe *Leishmania donovani* promastigotes with different morphologies (arrowhead) surrounded by cellular debris (*).

The first bioluminescent signals and parasites transcripts were detected as soon as 3 days p.i. (Fig. 1A). The parasite load was determined by RT-qPCR using a standard curve based on defined numbers of *L. donovani* parasites added to unparasitized tissues prior to nucleic acid extraction. Highly reproducible results were obtained for each sample performed in triplicate, and linearity was maintained over the range of template parasite numbers added to the brain. The parasite load in the brain was remarkably elevated as soon as day 3 p.i., estimated as 2.9 × 10^3^ in the cortex and 3.9 × 10^4^ in the base of the brain. The parasite load was quite constant throughout the study, presenting an estimated average between 900 and 12,000 parasites/brain. In addition, the parasite load was equally assessed by *in vivo* bioluminescence, with minimal individual variation, and the same kinetics’ profile observed by RT-qPCR (Fig. 1A).

In order to pinpoint the exact localization of the 2D bioluminescence signal detected through the cranium (Fig. 1B), we performed immunohistopathological analysis and a 3D bioluminescence assay combined with a micro–computed tomography (3D micro-CT). While histology and immunohistochemistry assays were not sensitive enough to evidence the parasite in brain sections, the detection and localization of bioluminescent parasites within the cranial cavity was possible by *in vivo* 3D micro-CT (Fig. 1C; Movie S1).

Following the parasite load detection in the brain using bioluminescence and RT-qPCR, we attempted to isolate live parasites from the brain by culturing fragments of brain tissues. We have successfully isolated parasites from both cortex (Fig. 1D) and base of the brain (Fig. 1E), at different time points. The base owned relatively more positive cultures than the cortex, with a peak of detection at day 21 p.i. All the parasites isolated from the brain presented morphologies and motilities equivalent to those from standard promastigote cultures (Fig. 1F). Similar positive cultures were obtained from fragments of brain tissues obtained from previously PBS-perfused animals (data not shown).

In order to minimize the possibility that the parasite load observed in the brain could result from the detection of parasites present in the cerebral circulation at the moment of euthanasia, we also performed parallel RT-qPCR on individual blood samples. In the blood, parasite burden reached an average of 2.7 × 10^1^ (± 1.4 × 10^1^) parasites/µL by day 3 p.i. This phase was followed by a rapid decrease at day 5 p.i closely reaching the positive threshold. Lastly, at day 30 p.i., the parasite load was considered negative (below the threshold), with 2.4 × 10^–1^ (± 8.8 × 10^–2^) parasites/µL (not shown). Considering that the mouse brain contains approximatively 20 µL of blood (50 µL/g of brain tissue)^28^, it is then unlikely that the RT-qPCR results in the brain would be influenced by parasites in the bloodstream.

### Parasite implantation and kinetics of infection in the periphery

**Figure 2:**
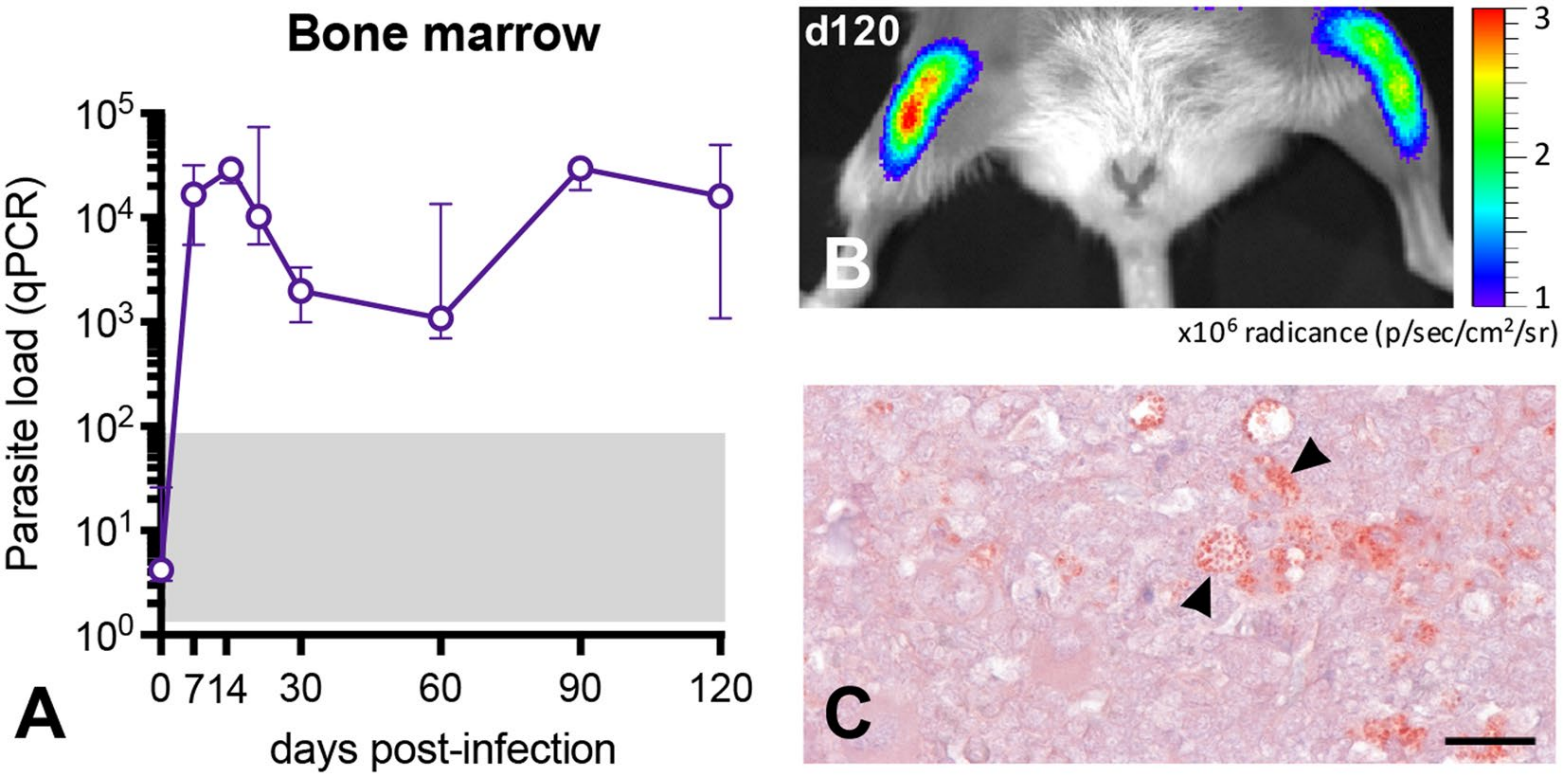
Parasite detection in the bone marrow of BALB/c mice infected with *Leishmania donovani*. (**A**) Kinetics of the parasite load in the bone marrow detected by RT-qPCR. The grey area corresponds to background signals in PCR. Data are represented as median and the interquartile range. (**B**) Representative image illustrating the 2D-bioluminescence analysis of the parasite detection in the femoral bone marrow at day 120 post infection. (**C**) Immunohistochemical detection of parasite amastigotes (arrowhead) in the femoral bone marrow. Immunoperoxidase, scale bar = 100 μm.

In order to determine the parasite load in other compartments, bone marrow, liver and spleen were evaluated. In the femoral bone marrow, two different phases of parasite load were observed; during the first phase, the parasite load rose up to 30,000 parasites over the first 14 days p.i., and then decreased slightly to reach 1,000 parasites at day 60. In the following phase, the parasite burden presented a further increase and reached more than 20,000 parasites at day 90 p.i. (Fig. 2A). The femoral bone marrow was not always suitable for *in vivo* bioluminescence evaluation, probably due to important barriers for light diffusion such as bones and muscles, giving positive results only when presenting an elevated parasite load (Fig. 2B), confirmed by immunohistochemistry (Fig. 2C).

Liver and spleen were included as reference compartments, since these organs are considered as the main targets for the parasite infection. The parasite load in the liver was observed throughout the study but it was remarkably high as early as day 7 p.i., reaching a peak of 9.3 × 10^7^ parasites at day 30 p.i., when a plateau was detected up to 120 p.i. (Figure S1A). The parasite load in the spleen has followed a similar profile as that of the liver (Figure S1B).

### Peripheral macrophages and neutrophils are recruited in the brain of *L. donovani*-infected mice

**Figure 3:**
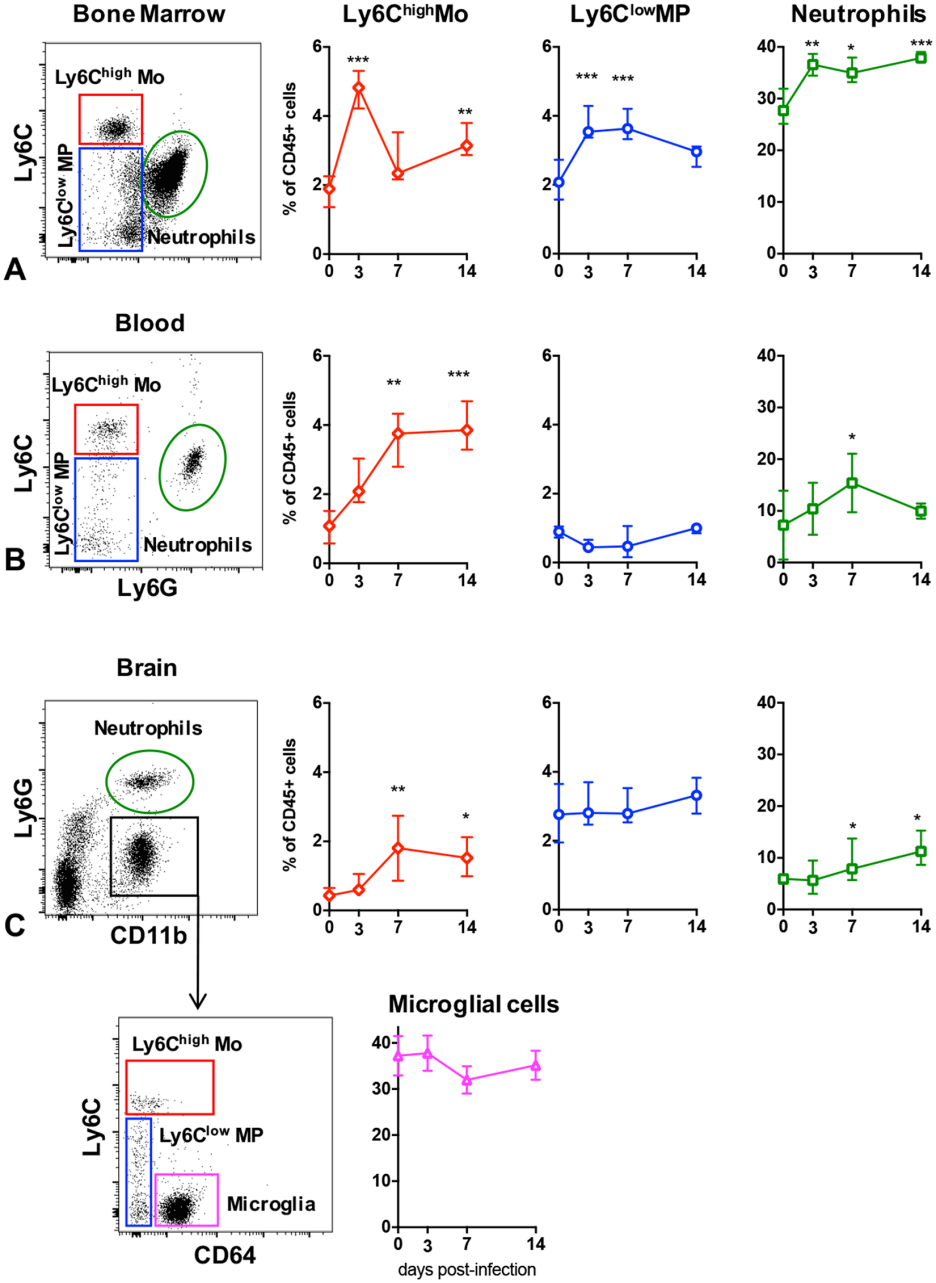
Myeloid cells dynamics in the bone marrow (**A**), blood (**B**) and brain (**C**) of BALB/c mice infected with *Leishmania donovani*. Dot plots and variations of inflammatory monocytes (CD45^+^CD11b^+^Ly6C^high^), patrolling anti-inflammatory monocytes (CD45^+^CD11b^+^Ly6C^low^), neutrophils and microglial cells at days 3, 7 and 14 p.i. Data are represented as median and the interquartile range. *Indicates P < 0.05.

In order to determine which cell type could act as a possible *Leishmania* carrier into the brain, we studied the dynamics of myeloid cell population in the bone marrow (Fig. 3A), blood (Fig. 3B) and brain (Fig. 3C) in the early phase of the infection (days 3, 7 and 14 p.i.). The bone marrow presented increased populations of CD45^+^CD11b^+^Ly6C^low^ mononuclear phagocytes (MP) and CD45^+^CD11b^+^Ly6C^high^ inflammatory monocytes from day 3 p.i., that raised in the circulation up to day 14 p.i. Neutrophils were elevated in the bone marrow throughout time, and up to day 7 p.i. in the peripheral blood. In the brain, both inflammatory monocytes and neutrophils were increased at days 7 and 14 p.i., with no changes concerning MP and microglial cells. Lymphoid cells were also evaluated, but no changes were observed in none of the compartments during this phase of the disease (data not shown).

### The brain environment becomes pro-inflammatory during *L. donovani* infection in mice

**Figure 4:**
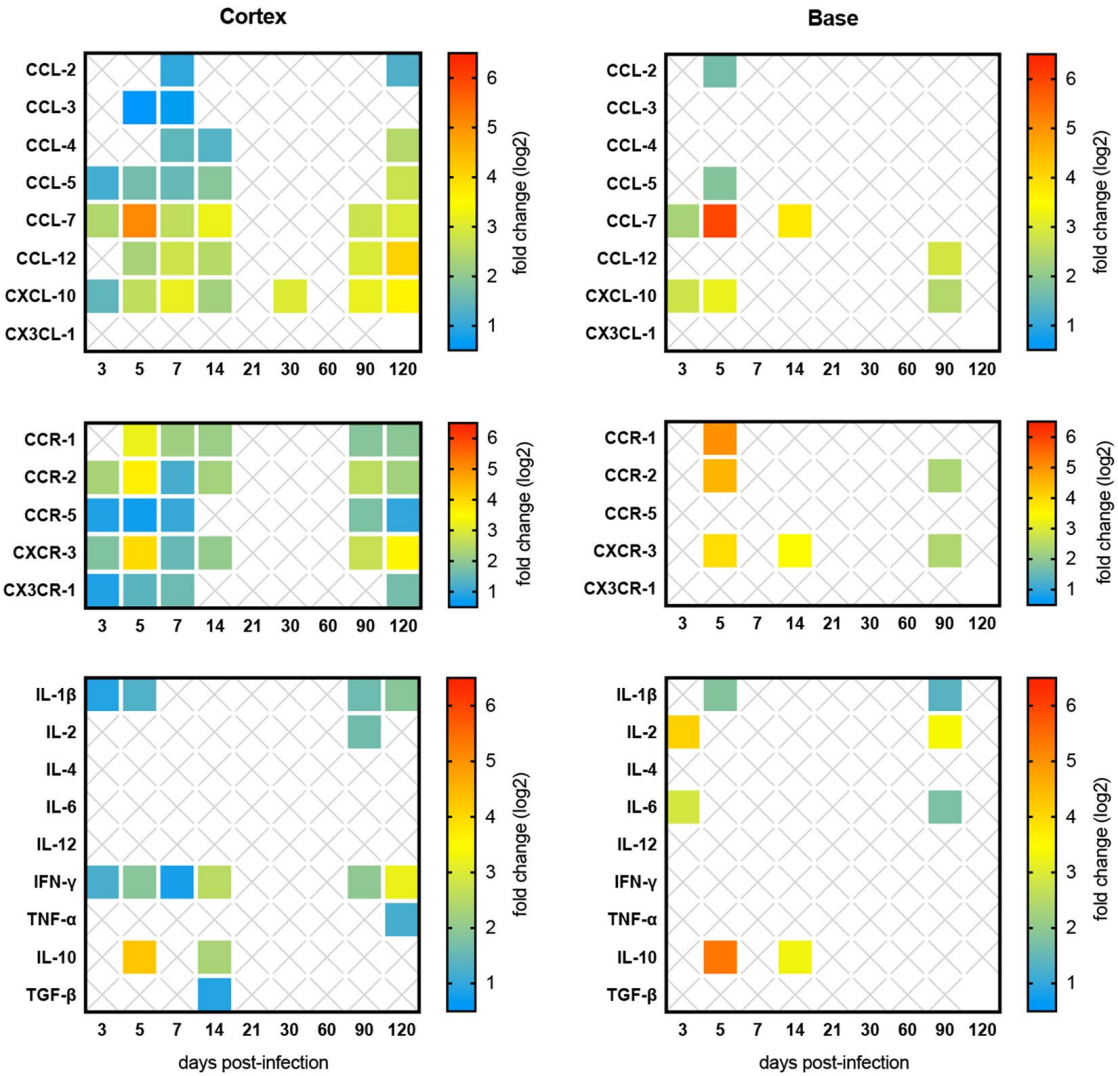
Inflammatory response assessment of BALB/c mice brains infected with *Leishmania donovani*. Relative gene expression of chemokines, chemokine receptors and cytokines in the cortex (left panel) and in the base (right panel). The results are expressed as fold change (up-regulation) for each time point post infection. Full-colored squares represent significant fold changes (P < 0.05).

Our analyses revealed a complex pattern of transcriptional modulations of pro-inflammatory factors indicating the triggering of two phases of inflammation, both cortex and base of the brain. The first phase (early inflammation phase) corresponded to day 3 until day 14 p.i.; and the second phase (named re-inflammation phase) was characterized by a second increase of inflammatory mediators by day 90 p.i. (Fig. 4). The comparison of the transcript abundances pointed out that the cortex presented higher number of up-regulated inflammatory mediators than the base.

We observed up-regulation of multiple chemokines and chemokine receptors in the cortex and the base during early and re-inflammation phases. The expression of chemokines was significantly up-regulated, especially CCL-7, with a peak at day 5 p.i., CCL-5, CCL-12 and CXCL-10, along with up-regulation of their respective receptors, notably CCR-1, CCR-2 and CXCR-3. Among all the up-regulated chemokines and chemokine receptors, CCL-7–CCL-12/CCR-2 and CXCL-10/CXCR-3 were the most noteworthy. The cytokine profile in the cortex and in the base presented the same dual-phased pattern, as for chemokines and chemokine receptors. At the early inflammation phase a mix of potent pro (IL-1β, IL-6, IFN- γ) and anti-inflammatory (IL-10, TGF-β) cytokines was observed, whereas, at the re-inflammation phase, only pro-inflammatory cytokines (IL-1β, IL-6 and TNF-α) were up-regulated (Fig. 4).

**Figure 5:**
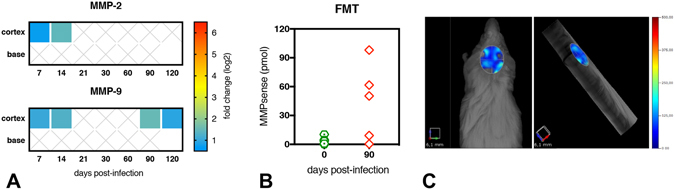
Transcript abundance and activity of matrix metalloproteinases (MMPs) in the brain of BALB/c mice infected with *Leishmania donovani*. (**A**) Relative gene expression of MMP-2 (upper panel) and MMP-9 (lower panel) in the cortex and in the base of infected mice brains. The results are expressed as fold change (up-regulation) for each time point post infection. Full-colored squares represent significant fold changes (P < 0.05). (**B**) *In vivo* detection of MMPs activity (including MMP-2, -3, -9 and -13) in the brain of mice at day 90 p.i. by quantitative fluorescence tomography (FMT) using the activatable fluorescent agent MMPsense 680^®^. (**C**) Tridimensional reconstruction of fluorescent tomography from a representative infected mouse exhibiting dorsal (left) and lateral (right) views, with a selected region of interest (ROI) delimitating the area of the brain, representative of 50.35 pmoles of MMPsense.

By means of RT-qPCR, we also checked the gene expression of the matrix metalloproteinases enzymes MMP-2 and MMP-9. MMPs could account for leukocyte invasiveness thus potentially increasing the population of monocytes harboring live amastigotes. As previously demonstrated for chemokines and cytokines, two phases were observed in the cortex of infected mice. The first phase (day 7 and day 14 p.i.) was characterized by the rapid increase of both MMP-2 and MMP-9 transcripts followed by a rapid decline. The second phase (from day 90 p.i. on) was delineated by a novel up-regulation of MMP-9. On the other hand, in the base of the brain, the gene expression of these enzymes presented no changes (Fig. 5A). Since MMPs are produced in an inactive latent form, we further evaluated the real activity of these enzymes *in vivo*. Using a 3D fluorescence molecular tomography (FMT) method and an activatable fluorescent agent, which is activated by MMPs, including MMP-2, MMP-3, MMP-9 and MMP-13, we detected extraordinary MMPs activity in 60% (3/5) of the infected mouse brains (Fig. 5B). This technique was sensitive enough to allow the anatomic localization, making possible the delimitation of regions of interest (Fig. 5C).

### The inflammatory response in the periphery is compartmentalized in *L. donovani*-infected mice

The bone marrow presented sporadic up-regulation of few chemokines and cytokines, with an interesting pattern of IFN-γ gene expression, similar to that observed in the brain, up-regulated within the first 21 days p.i. (2-fold) and after 90 days p.i. (2.7-fold). Th2 cytokines were upregulated in the early phase, including IL-10 (day 7 p.i.; 2.2-fold) and IL-4 (day 21 p.i.; 4.1-fold).

Liver and spleen were selected as the highest sites of infection and as different immune compartments to compare with the brain. The results indicated that the liver was the main target in this experimental model (Figure S1C). The gene expression of the pro-inflammatory cytokines IL-1β, IFN-γ and TNF-α along with the anti-inflammatory cytokine IL-10 and selected chemokines were significantly up-regulated throughout infection. On the other hand, in the spleen, no observable changes in the gene expression of inflammatory mediators occurred before 21 days p.i., except for IFN-γ, up-regulated at all time points, with the highest expression at 120 days p.i. (Figure S1D). The inflammatory involvement of the spleen becomes important after day 30 p.i., with predominance of pro-inflammatory mediators. The transcript abundance of MMP2 was not significantly increased during the infectious process neither in the liver or the spleen. Nevertheless, we noticed MMP-9 up-regulation in both organs, with significant values after day 14 p.i. (Figure S1C,D). By means of FMT, we detected increased MMPs activity (including MMP-2, -3, -9 and -13) in the liver of all infected mice (Figure S1E) and in the spleen of 4 out of 5 infected mice (Figure S1F). Additionally, with the three-dimensional reconstruction, we could clearly detect the fluorescent signals from the liver and from the spleen in the same image acquisition (Figure S1G).

## Discussion

**Figure 6:**
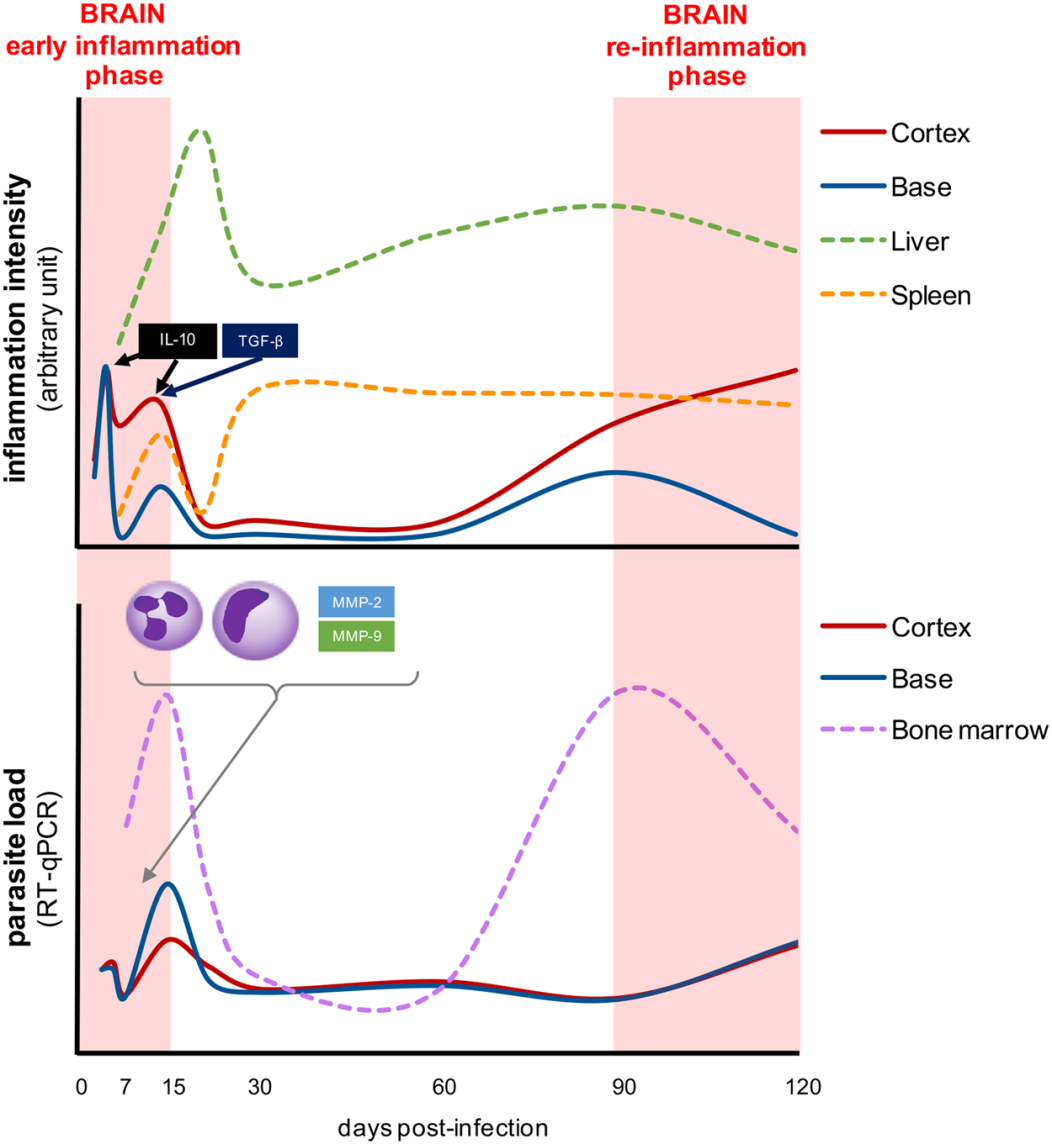
Inflammation dynamics in the brain of BALB/c mice infected with *Leishmania donovani*. In the upper panel, representative diagram of the inflammation intensity (arbitrary unity derived from the sum of all up-regulated cytokines, chemokines, chemokine receptors and matrix metalloproteinases) observed in the brain (cortex and base; full lines), and liver and spleen (dotted lines) of the infected mice. In the bottom panel, schematic parasite loads (medians) in the brain (cortex and base; full lines) and in the bone marrow (dotted line) of infected mice. Two similar waves can be observed in both panels: the first one, named early inflammation phase (light red shaded area), occurs in the first two weeks post-infection, when the peak of inflammation may be related to the arrival of parasites and inflammatory cells in the brain; whereas the second one, named re-inflammation phase (light red shaded area) occurs after 3 months of infection, where the novel increase of inflammatory mediators in the brain may be related to the novel increase of the parasite load in the bone marrow.

*Leishmania* donovani can infect and inflame the brain. Indeed, for the first time in mice, we were able to detect and to quantify live *Leishmania donovani* parasites in the brain, using a state-of-the-art approach combining *in vivo* 2D and 3D-bioluminescence imaging techniques, micro-computed tomography, RT-qPCR and parasite culturing, in a follow-up study. This is also the first longitudinal study to evaluate brain inflammation in experimental VL, which revealed a remarkable dynamics of inflammatory responses in the nervous environment, involving a multitude of cytokines, chemokines, enzymes and inflammatory cells (Fig. 6).

The parasite load in the brain of the *L. donovani* infected mice was noticeable and constant during the course of infection, with a worthy of note profile: the average number of parasites was estimated between 900 and 12,000, quite important considering the barriers and the immune privilege of the nervous environment. The presence of parasites and/or parasitized cells in the choroid plexus, meninges and cerebrospinal fluid in humans and dogs have already been the object of sporadic reports^17, 29, 30^, that contrast to unsuccessful attempts to visualize the parasites in the brain^31, 32^. This could be explained by the low number of parasites randomly distributed throughout the brain, or yet localized in small clusters in specific brain areas (as displayed in Fig. 1C).

Since *Leishmania* is an intracellular parasite in the vertebrate host, we may hypothesize that its dispersion towards the tissues possibly occurs via a ‘Trojan horse’ mechanism inside leukocytes^33^. Attempting to identify which cell type could act as a *Leishmania* carrier from periphery into the brain, we studied the dynamics of myeloid cell population in the bone marrow, blood and brain in the early phase of the infection (days 3, 7 and 14 p.i.). Of note, each compartment presented a specific cell profile. The bone marrow, the source compartment, presented increased populations of Ly6c^high^ inflammatory monocytes, Ly6c^low^ patrolling monocytes and neutrophils. Thus, bone marrow could act as a reservoir of parasitized cells, releasing them into the bloodstream in response to specific stimuli, and consequently allowing the infected cells to cross the BBB, similarly to some viruses^34^. In contrast, the blood, considered herein as a traffic compartment, presented a gradual increase of Ly6c^high^ monocytes and neutrophils, with a rather similar profile of that detected in the brain, the target compartment. In addition, parasitized macrophages - considered the main cells harboring the parasite during VL - but also neutrophils^35^, may contribute to brain disease, either carrying the parasites into the CNS, or by stimulating an inflammatory response. Nevertheless, as it has been recently demonstrated in the lungs^36^, we cannot differentiate the identified cells between marginated intravascular and parenchymal infiltrated leukocytes.

Whereas chemokines and chemokines receptors are key molecules to the recruitment of leukocytes into the inflamed sites^37^, cytokines are essential mediators to trigger or to suppress inflammation^38^. With a wide range of up-regulated chemokines, chemokine receptors and cytokines, the brain can easily be considered as a pro-inflammatory environment during *L. donovani* infection. More specifically, we have shown herein that two waves of inflammatory response take place in the brain, characterized by (1) an early inflammation phase from 3 to 14 days p.i., and (2) a re-inflammation phase starting at 90 days p.i., each one with specific features. The most striking finding is related to the upregulation of the chemokines/receptors CXCL-10/CXCR-3 and CCL-7/CCR-2 in the early-inflammation phase. These chemokines have been associated to the recruitment of leukocytes to the site of inflammation^37^, or to the increase of cell membrane permeability and neuronal apoptosis due to the increased expression of chemokine receptors by leukocytes and nervous cells as well^39^.

The present data demonstrate that CXCL-10 (also known as IFN-γ-induced protein 10 or IP-10) is the most persistent chemokine in the brain of *L. donovani* infected mice, which was shown to be involved in the neuropathogenesis of canine VL^40^ and in other protozoan infections, including cerebral malaria, toxoplasmosis and African trypanosomiasis^41–43^. Conversely, the release of CCL-7 (monocyte-chemotactic protein 3 or MCP-3) by astrocytes in the brain is related to the normal recruitment of perivascular macrophages, while its upregulation has been associated with inflammatory cells infiltration in viral infections, endotoxemia and stroke^44, 45^.

Completing the inflammatory mediators arsenal in the early inflammation phase, we detected MMP-2 and MMP-9 in the cortex, enzymes related to BBB disruption and leukocyte migration^46^. Similarly, in cerebral malaria, MMP-2 and MMP-9 were overexpressed as soon as 8 days p.i., with evidences of MMP-9 activation^47^. Interestingly, MMP-2 has already been associated to brain inflammation in canine VL^48^, but it may also play a role in the control of inflammation, specially by cleaving CCL-7^49^, which is concomitantly increased in the brain of infected mice.

Additionally, a mix of pro- (IL-1β and IFN- γ) and anti-inflammatory (IL-10 and TGF-β) cytokines was detected in the early inflammation phase in the brain. IFN-γ is a key cytokine to the induction of an immune response^50^ and IL-1β is considered a hallmark of brain inflammation capable of BBB disruption^51^, that can be efficiently downregulated by the overexpression of IL-10^52^. In a pro-inflammatory milieu, IL-10 is able to produce chemokine decoy receptors in monocytes and dendritic cells^53^ and consequently be responsible of the end of the early inflammation phase in the brain, with two highly significant successive peaks of up-regulation, observed on days 5 and 14 p.i. (Fig. 6). Similarly, TGF-β attenuates the destructive injuries due to the first wave of the inflammatory response in the brain^54^ maintaining the integrity of the BBB^55^ and reducing chemokines and adhesion molecules synthesis^56^. IL-10, in combination with TGF-β, seems to be powerful enough to completely suppress the gene expression of inflammatory mediators in the brain after 14 days of infection, but this is not an endless control, as reported also in a stroke model using IL-10 knockout mice, where IL-10 play a role in attenuation, but not in resolution of neuroinflammation^57^.

Three months after infection, a novel phase of inflammation arises, more evident in the cortex, which takes place in an environment free of anti-inflammatory mediators. The previous arsenal of chemokines and chemokine receptors found in the early inflammatory phase is again up-regulated in the re-inflammation phase, along with IFN-γ and two of the most potent pro-inflammatory cytokines, IL-1β and TNF-α, directly acting on chemokine production and BBB disruption^58^. Finally, MMP-9, an enzyme hallmark of inflammation^59^, was again up-regulated and the detection of activated MMPs *in vivo* evidenced their role in the neuroinflammation during VL, since no activation of MMPs occurred in non-infected mice, which may be related to BBB disruption and cell trafficking^46, 47, 59^. This second wave of inflammation in the brain was named “re-inflammation phase” since it does not seem to result from a chronification of an acute process (as for the liver and the spleen, depicted in Fig. 6), but rather due to a novel stimulus.

The two waves of inflammation observed in the gene expression of inflammatory mediators in the brain occur concomitantly to the parasite load peaks in the bone marrow (illustrated in Fig. 6), differently from the classic progressive growing pattern^60^. If the parasitized cells are released in the bloodstream, they could indeed reach the brain, specially under inflammatory conditions, when the replacement of meningeal and perivascular monocytes in the brain by hematogenous monocytes is enhanced^61^. Nevertheless, the parasite load in the brain is kept stable overtime, which does not support the premise of a new wave of parasites arrival. Comparable to intralesional parasites, the parasites in the brain may be turned into a semi-quiescent state, with slow rate of growing, thus allowing for a long-term expansion and persistence^62, 63^.

The re-inflammation phase in the brain is probably linked to the chronic systemic inflammation established in the periphery and not to the parasite itself ^64^. Both liver and spleen of the infected mice displayed a classical mixed inflammatory response against VL^65^, but is in the liver that the inflammation is promptly triggered, persistent and more intense. Recently, the concept of a liver-brain inflammation axis has called the attention of researchers. Peripheral cytokines are related to microglial activation, chemokine production and leukocyte recruitment into the brain in a model of liver inflammatory injury, leading including to behavioral changes^66^, which have also been observed in *Leishmania amazonensis* infected mice^67^.

Last but not least, the question about the potential link between BBB disruption, parasite burden and neuroinflammation with neurological clinical signs has currently not be solved. Case reports of human and canine VL underlined the sporadic occurrence of neurological clinical signs, with the presence, or not, of parasites in the brain^8, 17, 25, 29, 30, 68^. In all these circumstances, however, brain inflammation is a common finding, even in asymptomatic cases^31, 40, 69–71^. These data, along with the results presented herein, allow us then to hypothesize that brain inflammation is a key element during VL, and that it may precede the onset of neurological clinical signs.

Altogether, our studies demonstrated that *Leishmania donovani* is able to infect BALB/c mice and to persist in the tissues. More specifically, with this model, our study is pioneer to demonstrate brain involvement during infection, with detection of viable parasites throughout infection, from initial time points as early as three days after infection, up to four months. During this period, the brain presented a dual-phased and compartmentalized inflammatory response, with a profile of mediators that differed from those typically observed in the periphery^72^. Consequently, this study was crucial to include the brain in the list of organs affected by this parasite, particularly in endemic areas, and pave the way for further studies aiming to better understand the pathogenesis of VL and other chronic infectious diseases, focusing on the mechanisms underlying the arrival of pathogens and inflammatory cells into the brain, and on the brain effects under an inflammatory status in the periphery. Finally, since our experimental model shares some inflammatory mediators observed in the liver and the spleen with human and canine VL, we claim that the inflammatory environment and parasite detection observed in the brain of infected mice resembles what could happen in humans and in dogs.

## Methods

### Animals and parasites

Six-week-old female BALB/cByJRj mice were purchased from Janvier Laboratories (Le Genest-Saint-Isle, France), and handled under specific pathogen-free conditions, according to the institutional guidelines of the Central Animal Facility at Institute Pasteur. We used *Leishmania donovani* parasites (LD1S/MHOM/SD/00-strain 1 S) expressing the firefly luciferase gene^73^, which is a fully virulent strain^74^.

### Infection and *in vivo* imaging

Short-term cultures of *L. donovani* promastigotes were obtained from splenic amastigotes isolated from infected hamsters. The parasites were expanded until infectious metacyclic promastigotes from 9 days stationary phase cultures. Parasites were centrifuged at 1300 × g for 5 min at 20 °C and enriched metacyclic promastigotes were collected from the supernatant by centrifugation at 3500 × g for 10 min. Mice were infected with the standard dose of 5 × 10^7^ promastigotes in 150 μL of PBS by intraperitoneal route. Control mice were injected PBS. Before each sampling, we evaluated the infection using *in vivo* bioluminescence assays^73^. Briefly, at different time points following *Leishmania* inoculation, D-luciferin (122799, PerkinElmer), the luciferase substrate, was injected intraperitoneally at 500 mg/kg; the animals were anaesthetized in a 2.5% isoflurane atmosphere (Aerane^®^, Baxter SA) and placed in the imaging chamber of the IVIS™ Spectrum (PerkinElmer). 2D-bioluminescence images were captured and total photon emission, expressed in photons/s, was determined in a defined region of interest (ROI) using the Living Image software (PerkinElmer). Four independent kinetics of *in vivo* bioluminescence experiments were performed. 3D-micro–computed tomography using the IVIS™ Spectrum CT (PerkinElmer) was performed to provide the anatomic localization of the bioluminescence signals.

### Sampling

Following the 2D-bioluminescence analyses, representative infected mice, selected based on the bioluminescence values in the spleen^73^, were euthanized and biological samples were collected at specific post infection (p.i.) time points, according to the following schema. Control uninfected mice were collected accordingly.

**Table:** 

Sampling	Acute Phase						Chronic phase		
days p.i.	3	5	7	14	21	30	60	90	120
months p.i.	1						2	3	4

The entire brain was collected, including the leptomeninges, separated in two hemispheres and subsequently divided in two samples: (1) cortex, including the cerebral cortex and the hippocampus; and (2) base, which contains the brainstem and the diencephalon. We collected the liver, the spleen and the femoral bone marrow as representative samples of the systemic infection. Peripheral blood was also collected in tubes containing heparin.

### Parasite isolation from brain tissue

At different time points post-inoculation, paired cortex and base of three infected mice (totaling 21 infected mice) were collected in 1 mL of supplemented M199 medium and homogenized using the Precellys^®^ 24 System (Bertin Technologies). Three uninfected mice were collected as control. The volume of the homogenate was completed with supplemented M199 medium to 5 mL and centrifuged for 5 min at 150 × g. 500 µL of the supernatant was added into a 24-well plate and serial dilutions (10- and 100-fold) were made. The plates were incubated at 26 °C up to 14 days and observed in light microscopy to detect promastigote forms.

### RNA isolation and transcriptional analyses by quantitative PCR

At different time points following *Leishmania* infection, three infected mice were sacrificed (totaling 27 infected mice). A total of seven uninfected mice were used as control. Cortex and base of the brains were collected and homogenized in 1 mL of Trizol^®^ using the Precellys^®^ 24 System (Bertin Technologies). The bone marrow was flushed out from the femur using a syringe filled with PBS; the cells were pelleted by centrifugation at 1300 × g for 5 min at 4 °C, and homogenized in 1 mL of Trizol^®^. Due to volume limitation, we collected a total pool volume of 250 µL of blood in heparin only on days 3, 5 and 30 p.i., and homogenized in 750 µL of Trizol^®^. Livers and spleens were removed, disrupted and lysed in 5 and 3 mL of Trizol^®^ (Invitrogen), respectively, using tubes M and the gentleMACS^TM^ dissociator (Miltenyi Biotec).

RNA isolation was performed on the clear upper aqueous layer with the RNeasy Plus Mini kit (74134, Qiagen) according the manufacturer’s instructions. Evaluation of RNA quality was performed by optical density measurement using the NanoDrop spectrophotometer (Thermo Scientific) and their integrity were assessed using 2100 Bioanalyzer (Agilent Technologies) that allowed the calculation of an RNA integrity (RNAi) number. Total RNAs were reverse transcribed to first strand cDNA using random hexamers (11034731001, Roche Diagnostics), a set of dNTPs (10297-018, Invitrogen) and Moloney Murine Leukemia Virus Reverse Transcriptase (MMLV-RT, 28025013, Invitrogen).

PCR was performed in a final volume of 11 μL per reaction in 384-well PCR plates (Thermo Scientific) using a thermocycler (7900HT fast real time PCR system, Applied Biosystems) in triplicates. Briefly, 2 μL of cDNA (20ng) was added to 9 μL of a master mix containing 5 μl of QuantiTect SYBR Green Kit (Qiagen) and 4 μL of nuclease-free water with primers at a final concentration of 1 μM (Table S1). The amplification conditions were as follows: 95 °C for 15 min, 45 cycles of 95 °C for 10 s, 54 °C for 25 s and 72 °C for 30 s; followed by a melt curve, from 60 °C to 95 °C. The mouse gene targets were selected for quantifying host inflammatory mediators transcripts in the brain (cortex and base), liver, spleen and bone marrow (Table S1).

For normalization calculations, candidate control genes were tested with the geNorm algorithm^75^. The pairs ldha (lactate dehydrogenase A)/tbp (TATA box-binding protein) and l19 (ribosomal protein L19)/rpIIe (RNA polymerase E) were selected as the most stable reference genes for the cortex and base of mice brains, respectively. The pairs l19/rpIIe, ldha/l19 and tbp/rpIIe and were selected as reference genes for the bone marrows, livers and spleens respectively.

### Quantification of parasites by RT-qPCR

Serial 10-fold dilutions of parasites were added either to brains (from 10^5^ to 10^0^), bone marrows (from 10^6^ to 10^1^), livers (from 10^8^ to 10^3^) and spleens (from 10^8^ to 10^3^) recovered from naïve mice. Total RNAs were further extracted and processed for RT-qPCR as described above. The *Leishmania* gene target (*ssrRNA*) was selected for quantifying the number of parasites as previously described on murine cDNAs^73^. A linear regression for each standard curve was determined (quantification of *Leishmania* parasites against the relative expression of *ssrRNA* values).

### Flow cytometry

We evaluated the myeloid and lymphoid cells populations in brain, bone marrow and blood at early time points p.i. by flow cytometry. Mice were infected at days -3, -7 and -14 and they were sacrificed at day 0, in order to have age-matched mice with 3, 7 and 14 days p.i. Four infected mice for each time point (totaling 12 infected mice) and four uninfected ones were sacrificed and samples were collected. 100 µL of blood were collected in heparinized tubes and placed in a 96-wells plate. The femur was collected in 1 mL of PBS containing 2 mM of EDTA and 0.5% (v/v) of fetal calf serum; the bone marrow was extracted by flushing the buffer into the medullary cavity using a 25 G needle after cutting the epiphysis. After homogenization, 100 μL of cell suspension was placed in a 96-wells plate. Regarding the brain, the whole organ was collected in 4 mL of PBS containing 2 mM of EDTA and 0.5% (v/v) of fetal calf serum and dissociated using a cell strainer of 70 μm. The cell suspension was centrifuged at 300 × g for 10 min at 4 °C and the pellet was resuspended in 8 mL of 35% (v/v) of Percoll gradient (P1644, Sigma-Aldrich). The mixture was centrifuged at 400 × g for 20 min at 20 °C, the pellet was collected, washed in PBS-EDTA, resuspended in 200 µL of PBS-EDTA and placed in a 96-wells plate.

The 96-wells plate containing all the samples was centrifuged at 300 × g for 5 min at 4 °C, the supernatant was removed and 50 μL of a cocktail of antibodies containing: anti-CD11b (clone M1/70), anti-Ly6C (clone AL-21), anti-Ly6G (clone 1A8), anti-CD45 (clone 30-F11) (Becton Dickinson), anti-CD64 (clone × 54–5/7.1.1) (BioLegend) was added and incubated for 20 min at 4 °C. The cells were washed in PBS and the red blood cells were lysed by washing three times with ACK (Ammonium-Chloride-Potassium) Lysing Buffer. The plate was centrifuged and the cells were resuspended in 4% (w/v) of paraformaldehyde. Flow cytometry was performed with the flow cytometer Fortessa-X20 (Becton Dickinson) and DIVA^®^ Flow Cytometry acquisition software and was analysed with FlowJo software (Tree Star, Inc).

### *In vivo* activity of matrix metalloproteinases enzymes

We evaluated the activity of the matrix metalloproteinases enzymes by quantitative fluorescence molecular tomography (FMT). At 90 days p.i., five infected mice and five uninfected ones were injected intravenously with 2 nmol of MMPsense 680 (NEV10126, PerkinElmer), a fluorescent agent activated by matrix metalloproteinases, including MMP-2, -3, -9 and -13. Then, 30 hours after injection, the animals were anaesthetized in a 2.5% isoflurane atmosphere (Aerane^®^) and placed in the imaging chamber of the FMT 2500^TM^ system (VisEn Medical), where 3D-fluorescence data were acquired. The collected data were reconstructed using the TruQuant software (PerkinElmer), three-dimensional regions of interest (ROIs) were delimited for each brain, liver and spleen, and the fluorescence values were automatically converted in pmols according to internal standards, after applying a threshold equal to 2-times the mean fluorescence value (nM) detected in the control uninfected mice^76^.

### Histology and Immunohistochemistry

At different time points post-inoculation according to the scheme in item 2.3, brain, liver, spleen and femur were collected. The femur was decalcified and all samples were embedded in paraffin. Four μm–thick sections were stained with hematoxylin and eosin (HE) or used for immunohistochemistry. A rabbit polyclonal antibody (1:3500) was used to detect *Leishmania* parasites on a Bond III® immunostainer (Leica).

### Statistical analyses

The comparison between infected and control groups was performed by Mann-Whitney or Kurskal-Wallis tests. Values of P < 0.05 were considered statistically significant. Data were expressed as the median and the interquartile range. For qPCR, the variations in the gene-expression were calculated as the n-fold change in expression in the organs from the infected mice compared to the organs of the uninfected ones. The relative expression software tool (*REST* ^*©*^ *-MCS)* was used for determining group wise comparison and statistical analysis of relative expression results in real-time PCR^77^.

### Ethical statement

All animal experiments were conducted in accordance to the project registered under number 2013–0047 and approved by the Institut Pasteur Ethics Committee (CETEA) on November 12, 2014 in accordance to the European legislation/guidelines EU 2010/63.

### Data availability

All data generated or analysed during this study are included in this published article (and its Supplementary Information files).

## Electronic supplementary material

Supplementary Information

Movie S1
