# Supplementary material for: Unveiling Cerebral Leishmaniasis: parasites and brain inflammation in Leishmania donovani infected mice
Source: Sci Rep. 2017 Aug 16;7:8454. doi: 10.1038/s41598-017-09085-5 (PMC5559479; doi:10.1038/s41598-017-09085-5)
Supplement: Supplementary file 1 — Supplementary Information [file 41598_2017_9085_MOESM1_ESM.pdf]

UNVEILING CEREBRAL LEISHMANIASIS:

parasites and brain inflammation in *Leishmania donovani* infected mice

Guilherme D. Melo, Sophie Goyard, Laurence Fiette, Alexandre Boissonnas, Christophe Combadiere, Gisele F. Machado, Paola Minoprio, Thierry Lang

TABLE S1 – Primers sequences used for RT-qPCR and reaction efficiency values (E) for each target.

Target	Forward (5' – 3')	Reverse (5' – 3')	E
Leishmania ssrRNA	CCATGTCGGATTTGGT	CGAAACGGTAGCCTAGAG	2.04
CCL-2 (MCP-1)	CAAGAAACGCAAGCGG	ACGGGATCTGAAAGACG	1.76
CCL-3 (MIP-1α)	ACCACTGCCCTTGCTGTTC	TCTGCCGGTTTCTCTTAGTCAG	1.95
CCL-4 (MIP-1β)	CCAATGGGCTCTGACC	ACTCCAAGTCACTCATGT	2.13
CCL-5 (RANTES)	GCTGCCCTCACCATCATCC	GTATTCTTGAACCCACTTCTTCTCTG	1.82
CCL-7 (MCP-3)	CCAATGCATCCACATGCTGC	GCTTCCCAGGGACACCGAC	2.11
CCL-12 (MCP-5)	AGTCCTCAGGTATTGGC	ACTTCTCCTTGGGGTCA	2.17
CCR-1	CATTTCCCCTACAAGAGC	CAAATATCAGACGCACGG	2.00
CCR-2	AGCACTTAGACCAGGC	CCAACCGAGACCTCTT	1.93
CCR-5	CTAGACCAGGCCATGC	CCTGTGGATCGGGTAT	1.97
CX3CL-1 (fractalkine)	CCTCACTAAAAATGGTGGCAAG	ATGTCAGCCGCCTCAAAAC	1.81
CX3CR-1	CAACCCCTTTATCTACGC	GCTTGTGTAGTGAGTGAAAC	2.04
CXCL-10 (IP-10)	GCAACTGCATCCATATCG	GGATTCAGACATCTCTGCT	1.89
CXCR-3	CAGCCTGAACTTTGACAGAACC	GCCGAAAACCCACTGGAC	1.95
IFN-γ	CTTCTTCAGCAACAGCAAGG	TGAGCTCATTGAATGCTTGG	1.83
IL-1β	AGGCAGGCAGTATCAC	CACACCAGCAGGTTATC	1.84
IL-2	AGGAACCTGAAACTCCC	AGTCCACCACAGTTGC	2.04
IL-4	GGAGCCATATCCACGG	AAGCCCTACAGACGAG	1.88
IL-6	ACAACGATGATGCACTT	CTTGGTCCTTAGCCACT	2.00
IL-10	CTGGACAACATACTGCTAACCGAC	ATTCATTCATGGCCTTGTAGACACC	1.99
IL-12p35	GGCCACCCTTGCCCTCCTA	GGGCAGGCAGCTCCCTCTT	1.91
TGF-β1	GCGGACTACTATGCTAAAGA	GTAACGCCAGGAATTGT	1.84
TNF-α	CATCAGTTCTATGGCCC	GTGAGGAGCACGTAGT	1.92
MMP-2	CGCTCAGATCCGTGGTGA	CGCCAAATAAACCGGTCCTT	2.19
MMP-9	AAAACCTCCAACCTCACGGA	GCGGTACAAGTATGCCTCTGC	2.12
L19	TACTGCCAATGCTCGG	AACACATTCCCTTTGACC	1.93
LDHA	AACCCTCAAGGACCAG	CAAGCTCATCCGCCAA	2.21
RPIIE	AAGATCCGCAAGACGA	GGGAAGAACACAAACATCTG	2.04
TBP	CCTATGACCCCTATCACT	GTCCGTGGCTCTCTTAT	1.86

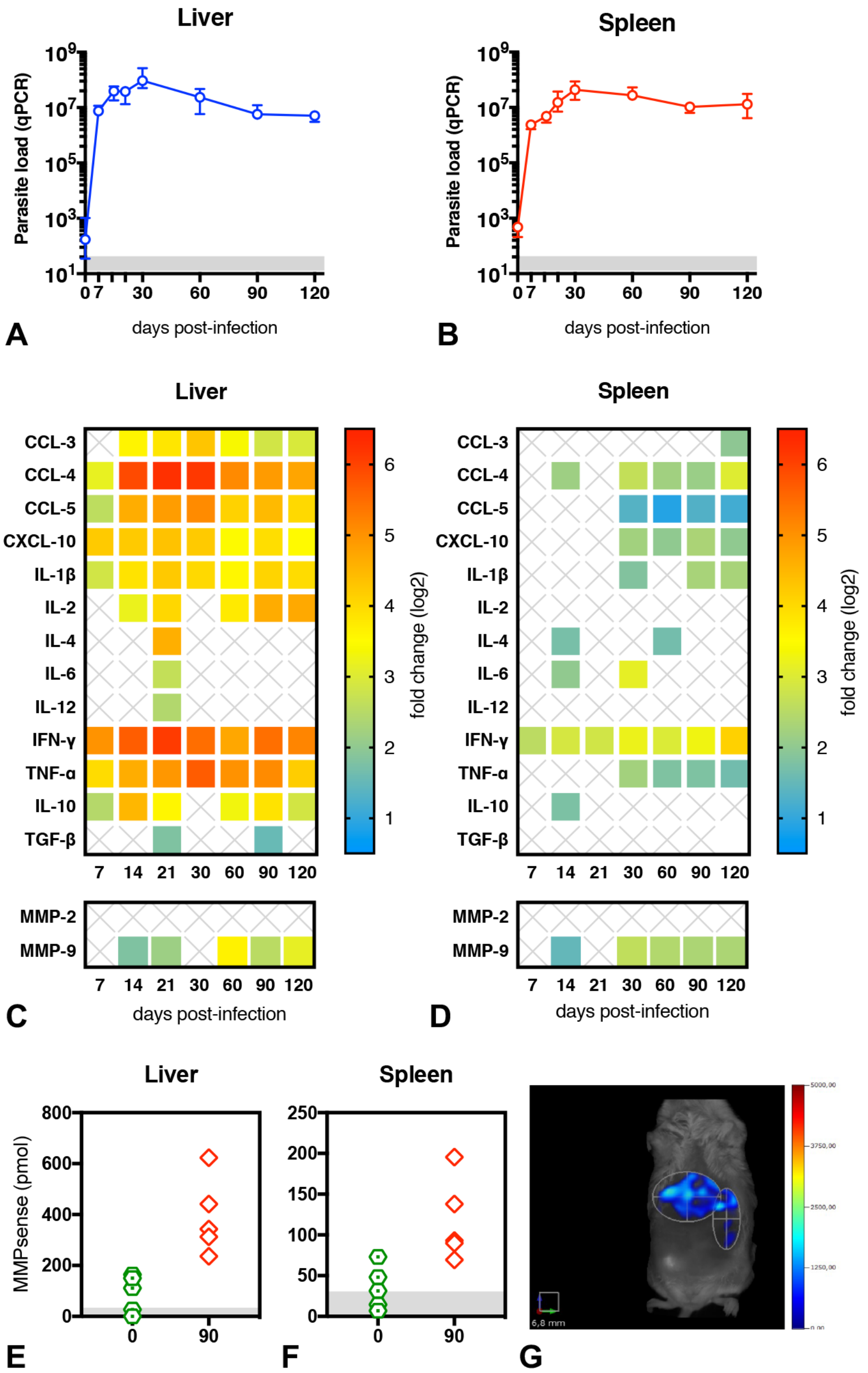

FIGURE S1. Parasite load and inflammatory response assessment in the liver and in the spleen of BALB/c mice infected with *Leishmania donovani*. (**AB**) Parasite load detected by RT-qPCR in the liver (A) and in the spleen (B). The grey area corresponds to background signals. Data are represented as median and the interquartile range. (**CD**) Relative gene expression of chemokines, cytokines and matrix metalloproteinases and in the liver (C) and in the spleen (D). The results are expressed as fold change (up-regulation) for each time point post infection. Full-colored squares represent significant fold changes ($P<0.05$). (**EF**) *In vivo* detection of MMPs activity (including MMP-2, -3, -9 and -13) in the liver (E) and spleen (F) of mice at day 90 p.i. by FMT using the activatable fluorescent agent MMPsense 680®. The grey area corresponds to background signals. (**G**) Tridimensional reconstruction of FMT from a representative infected mouse showing a ventral view, with selected region of interests (ROI) delimitating the areas of the liver and the spleen.

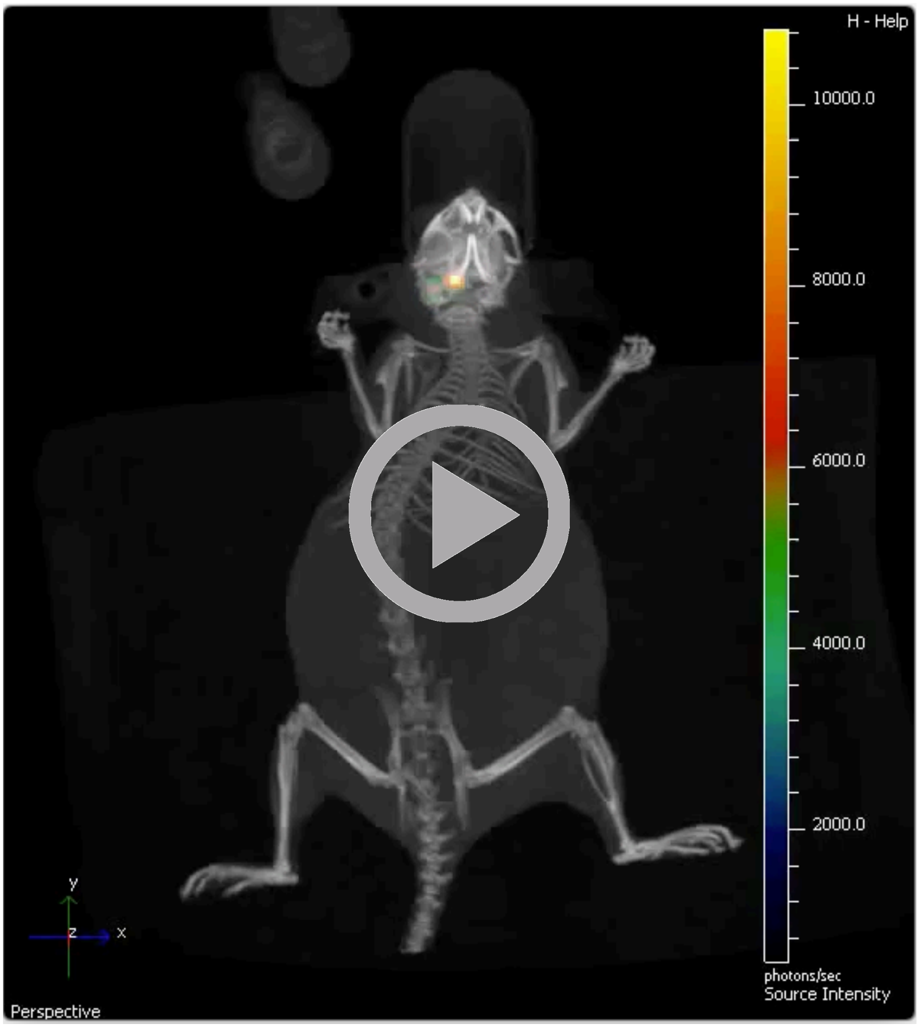

MOVIE S1. *In vivo* parasite detection in the brain of BALB/c mice infected with *Leishmania donovani*, Related to Figure 1. *In vivo* tridimensional micro-computed tomography to localize the bioluminescence signals within the brain. Representative tridimensional reconstruction of an infected mouse at 120 days post-infection exhibiting two bioluminescent foci within the cranial cavity.
